# Malignant and metastatic giant cell tumors of bone; clinical course of primary or secondary malignant and pulmonary metastatic variants

**DOI:** 10.1016/j.jbo.2025.100728

**Published:** 2025-11-24

**Authors:** Floortje G.M. Verspoor, Gitte G.J. Krebbekx, Mylene J.C. Duivenvoorden, Vaiyapuri Sumathi, Scott Evans

**Affiliations:** a Royal Orthopaedic Hospital, Oncology Department, Birmingham, United Kingdom; b University Hospitals of Birmingham, Birmingham, United Kingdom; c Amsterdam UMC, University of Amsterdam, Department of Orthopaedic Surgery and Sport Medicine, Amsterdam Movement Sciences, Amsterdam, the Netherlands

**Keywords:** Benign pulmonary metastatic GCTB, Primary malignant GCTB, Secondary malignant GCTB, Secondary osteosarcoma, Survival, Giant cell tumor of bone

## Abstract

**Aims:**

Giant cell tumor of bone (GCTB) is a rare, locally aggressive, intermediate-grade neoplasm. While typically benign, GCTB can exhibit atypical behavior, including indolent pulmonary metastases and malignant transformation. This study characterizes malignant and metastatic GCTB, distinguishing primary malignant GCTB (PM-GCTB), secondary malignant GCTB (SM-GCTB), and secondary osteosarcoma, and evaluates their outcomes.

**Methods:**

A retrospectively review of 520 patients with suspected GCTB treated between 1985–2021 was performed, with malignant cases followed through 2025. Diagnosis was confirmed histologically and, where available, by H3F3A/H3F3B mutation testing. PM-GCTB was defined as a de novo high-grade sarcoma. SM-GCTB and secondary osteosarcoma were defined as malignant transformations of previously benign, histologically proven GCTB. Benign pulmonary metastases were analyzed separately. Outcomes were descriptively assessed.

**Results:**

Twelve patients (2.4 %) had malignant GCTB: five PM-GCTB (1.0 %), five SM-GCTB (1.0 %), and two secondary osteosarcoma (0.4 %). Three patients (0.6 %) developed histologically benign pulmonary metastases with indolent behavior. Three SM-GCTB patients transformed within one year despite benign baseline pathology, indicating true rapid malignant progression rather than diagnostic error. Median latency to malignant transformation was 7.5 years for SM-GCTB and up to 35 years for secondary osteosarcoma. SM-GCTB showed the highest disease-specific mortality (80 %), compared to PM-GCTB (20 %) and secondary osteosarcoma (0 %). None malignant transformations occurred following denosumab or radiotherapy.

**Conclusion:**

Three aggressive GCTB variants were identified: benign pulmonary metastatic GCTB, PM-GCTB, and secondary malignant transformation. SM-GCTB and osteosarcoma arise from benign GCTB but differ in morphology and prognosis. Because malignant transformation is exceptionally rare and symptomatic, a patient-centered, symptom-driven follow-up strategy is preferred over routine lifelong radiologic surveillance.

## Highlights

• Benign GCTB may develop indolent pulmonary metastases (deposits).
• GCTB can transform into secondary malignant GCTB or secondary osteosarcoma.
• Some benign GCTB can show rapid malignant transformation.
• Malignant GCTB variants differ significantly in latency and prognosis.
• No malignant transformations occurred after denosumab or radiotherapy.

## 1 Introduction

Giant cell tumors of bone (GCTB) are rare, intermediate-grade, locally aggressive tumors that typically arise in the epiphysis of long bones, most often around the knee joint [[1], [2], [3]]. They primarily affect skeletally mature young adults and present with progressive pain, swelling, or pathological fractures [4]. These lesions can impair joint function and quality of life [5]. Although histologically benign, GCTB has a recurrence rate of up to 30 %, influenced by tumor characteristics, location, and treatment strategy [6,7].

While most GCTB behave benignly, a small subset exhibit atypical or aggressive behavior. Such cases include [1] pulmonary metastases that remain histologically benign, [2] primary malignant GCTB (PM-GCTB), representing a de novo high-grade sarcoma at diagnosis, and [3] secondary malignant transformations (SM-GCTB or secondary osteosarcoma) developing in previously benign lesions. However, the incidence and behavior of these variants remain unclear due to inconsistent terminology and a lack of diagnostic consensus [8,9]. Accurate distinction between these entities can be challenging, particularly in limited curettage samples, although true malignant transformation should be considered when recurrence shows sudden change in clinical or radiologic behavior [10,11].

Radiologically, GCTB appears as an eccentric, osteolytic lesion with non-sclerotic margins [12]. Campanacci classified GCTBs into three radiographic grades based on cortical involvement and soft tissue extension [2]. Their appearance may mimic other bone lesions such as aneurysmal bone cysts, brown tumors, or malignancies [12].

Histologically, GCTB contains neoplastic mononuclear stromal cells admixed with uniformly distributed numerous osteoclast-like giant cells. The neoplastic cells characteristically harbor H3F3A mutations (typically G34W) and express RANKL, which is targeted by denosumab therapy [13,14]. Treatment generally includes intralesional curettage with or without local adjuvants (e.g., phenol, cryotherapy, denosumab), or wide resection depending on anatomical and clinical factors [2,6,14].

The recognition of H3F3A/B mutations has improved diagnostic accuracy and allows more reliable distinction between GCTB and other giant-cell-rich sarcomas. Nevertheless, malignant GCTB remains diagnostically complex. PM-GCTB arises de novo as a high-grade sarcoma, whereas SM-GCTB and secondary osteosarcoma represent malignant transformations within prior benign lesions [[10], [11], [15]]. In contrast, benign pulmonary metastases represent a separate entity with unpredictable but usually indolent clinical behavior [9], [16].

Despite reported cases, systematic data comparing these malignant and metastatic variants remain scarce. Understanding their presentation, treatment, and outcomes is critical to improve early diagnosis and to optimize long-term surveillance.

Therefore, this retrospective study analyzed all patients with GCTB treated at a high-volume sarcoma center between 1985 and 2021, with the aim to characterize benign metastatic, primary malignant, and secondary malignant forms, to clarify their diagnostic features, clinical course, and prognostic implications.

## 2 Methods

### 2.1 Study design and population

We retrospectively analyzed 520 patients with histologically confirmed or initially suspected giant cell tumor of bone (GCTB) who presented at our tertiary bone referral center between January 1985 and November 2021 with follow-up data of malignant GCTB updated until June 1, 2025. Ethical approval was obtained from the local audit and research committee (Ref: W22_022 # 22.051). Because this inclusion period spans major diagnostic developments, histopathologic criteria and molecular tools evolved substantially. Since 2017, H3F3A/H3F3B sequencing or immunohistochemistry has been routinely applied; earlier cases were re-reviewed whenever archival tissue was available.

Patients were identified from a prospectively maintained institutional database. All data were anonymized prior to analysis. Inclusion criteria were histologically confirmed GCTB. After exclusion of 14 giant-cell-rich osteosarcomas, 506 eligible patients remained.

### 2.2 Data collection and definitions

Two investigators (FGMV, SE) independently reviewed medical records for demographics, histology, treatment, and follow-up. Survival status was classified as: no evidence of disease (NED), alive with disease (AWD), died of disease (DOD), or died of other causes (DOOD). Histopathological diagnoses of biopsies were confirmed by an experienced musculoskeletal pathologist (VS), with mutational testing performed when tissue permitted. All malignant or equivocal cases were re-evaluated by an experienced bone pathologist (VS) using updated WHO 2020 criteria and H3F3A/B status.

Malignant and metastatic categories were defined as:

• Primary malignant GCTB (PM-GCTB): a primary high-grade sarcoma arising de novo, without a prior benign GCTB lesion.
• Malignant transformation of a previously benign GCTB.
  o Secondary malignant GCTB (SM-GCTB): a sarcomatous transformation arising at the site of a previously benign, histologically confirmed GCTB, maintaining some morphological resemblance to the original lesion.
  o Secondary osteosarcoma: a transformation of a formerly benign GCTB into a conventional high-grade osteosarcoma, characterized by production of malignant osteoid.
• Benign pulmonary metastases: histologically benign GCTB deposits in the lungs without sarcomatous change.

Three cases underwent diagnostic reclassification after expert re-review: two cases originally labeled malignant GCTB were identified as secondary osteosarcoma, and one case initially labeled PM-GCTB was reclassified as SM-GCTB based on the presence of a preceding benign lesion.

To assess therapy-related risk, denosumab- and radiotherapy-exposure were recorded for the entire cohort of benign GCTB (denosumab n = 130; radiotherapy n = 8); none of the malignant transformations occurred in these patients.

### 2.3 Statistical analysis and visualization

**Fig. 1:**
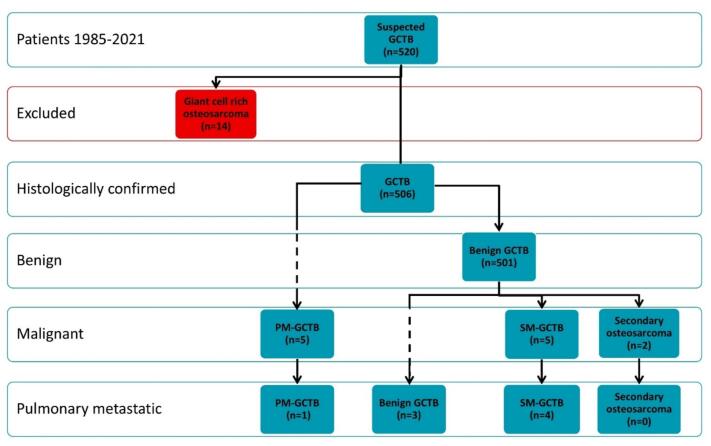
Flowchart of consecutive patients with Giant Cell Tumors of Bone. Primary malignant GCTB (PM-GCTB), secondary malignant GCTB (SM-GCTB), secondary osteosarcoma, benign pulmonary GCTB metastases (deposits) without sarcomatous change.

**Fig. 2:**
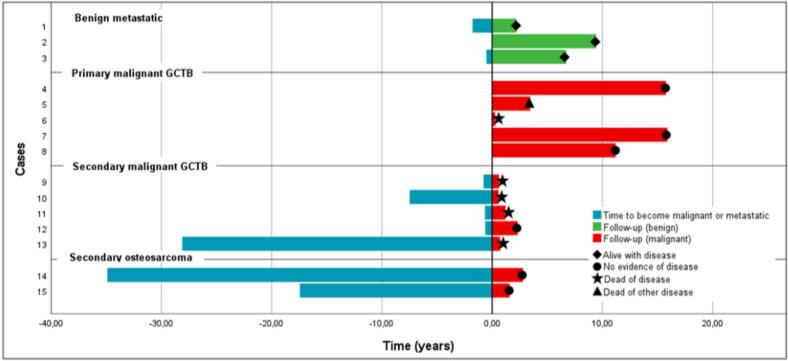
Swimmer’s Plot of Disease course and follow-up in Malignant and Metastatic Giant Cell Tumors of Bone.

Descriptive statistics were calculated using SPSS v28 (IBM Corp., Armonk, NY). Categorical variables were reported as frequencies (%), continuous variables as medians (IQR). Due to small sample sizes and data heterogeneity, the analyses were exploratory in nature. Group comparisons were performed using pairwise Fisher’s exact tests with simulated p-values for binary outcomes, and the Kruskal–Wallis test for non-normally distributed ratios. A p-value < 0.05 was considered statistically significant. Diagnostic subtypes, latency, and outcomes were illustrated in Fig. 1 (flowchart) and Fig. 2 (swimmer plot).

## 3 Results

### 3.1 Cohort characteristics

After exclusion of 14 giant cell-rich osteosarcoma cases, 506 patients with histologically confirmed GCTB were analyzed. The median age at diagnosis was 34 years (IQR 25–47), with no significant age differences between benign, primary malignant (PM-GCTB), secondary malignant (SM-GCTB), or metastatic groups (p = 0.39). Sexes were equally distributed (p = 0.08), males accounted for 49 % of patients. Benign non-metastatic tumors were most commonly located in the femur (31 %), tibia (26 %), radius (9 %), pelvis (7 %), fibula (6 %), humerus (5 %), and hand (5 %), without significant variation between groups (p = 0.63). Primary malignant (PM-GCTB), secondary malignant (SM-GCTB), or benign metastatic GCTB were located at similar locations which are specified in Table 1. The anatomic distribution of malignant and metastatic variants mirrored that of benign GCTB, supporting their shared origin rather than site-specific behavior.

**Table 1:** Characteristics of Giant Cell Tumors of Bone patients.

Giant cell tumors of bone	Benign	Benign Pulmonary Metastatic	Primary malignant (PM-GCTB)	Secondary malignant (SM-GCTB)	Secondary osteosarcoma	P-value
N (%)	501 (97)	3 (0.6)	5 (1.0)	5 (1.0)	2 (0.4)	
Median age at diagnosis in years (IQR)	34 (25–47)	29 (19–29)	46 (23–70)	28 (25–57)	18 and 23	0.39 0.92^x^
Gender, male N(%)	244 (49)	2 (67)	5 (100)	3 (60)	2 (100)	0.08 0.06^x^
Location N (%)						
Femur	156 (31)	1 (34)	3 (60)	2 (40)		0.63
Tibia	130 (26)		1 (20)			0.77^X^
Radius	46 (9)	1 (33)	1 (20)	2 (40)	2 (100)	
Pelvic	36 (7)	1 (33)				
Fibula	31 (6)			1 (20)		
Humerus	26 (5)					
Hand	23 (5)					
Other	38 (10)					
Unkown	15 (3)					
Median total FU time (IQR)	4 years (2–7)	7.2 (4.0–9.4)	11.2 (1.9–15.8)	2.94 (1.6–18.5)	19.0 & 36.4	0.21 ^X^
FU time from benign to ‘benign metastatic’ or ‘malignant’ in years Median (IQR)	NA	3.2 (1.3–4.4)	NA	7.5 (0.6–7.5)	17.4 & 34.9	0.44 ^X^
FU time after transformation in years Median (IQR)	NA	6.7 (2.2–9.4)	11.2 (1.9–15.8)	0.7 (0.6–1.8)	1.6 & 2.8	0.13 ^X^
Denosumab N(%)	129 (26)	1 (33)	0	0	0	0.04 ^X^
RTx N(%)	7 (0.1)	1 (33)	0	0	0	0.04 ^X^
Chemotherapy N(%)	NA	1 (33)	2 (40)	4 (80)	2 (100)	0.63 ^X^
Recurrence N(%)	110 (22)	0	1 (20) *	2 (40) *	0	0.84 ^X^
Metastases N(%)	0	3 (100)	1 (20)	4 (80)	0	0.02 ^X^

Other= Clavicle (n = 2), Foot (n = 15), Rib (n = 7), Toe (n = 1), Ulna (n = 12), Vertebra (n = 1). FU duration reflects available follow-up and does not indicate routine surveillance intervals.

NA = not applicable.

* = After development into malignant or benign metastatic disease.

x = P-value excluding benign GCTB.

Histopathologic confirmation was based on biopsy and/or resection specimens in all patients. For cases diagnosed before 2017, slides were re-reviewed, and H3F3A/H3F3B mutation analysis was performed where archival tissue allowed. All malignant and metastatic diagnoses were confirmed by an expert musculoskeletal pathologist using current WHO 2020 criteria.

### 3.2 Tumor subtypes and incidence

Among 506 patients, 501 (99 %) were benign at presentation, and five (1 %) represented PM-GCTB. Within the benign cohort, three patients (0.6 %) later developed benign pulmonary metastatic GCTB, and seven (1.4 %) experienced secondary malignant transformation (five evolving into SM-GCTB and two into secondary osteosarcomas) (Fig. 1).

### 3.3 Benign non-metastatic GCTB

The majority of benign GCTB were treated with intralesional curettage (74 %) using local adjuvants such as phenol, cryotherapy, or polymethylmethacrylate, and 26 % underwent en-bloc resection.

Overall local recurrence was 22 %, consistent with previous literature. Median follow-up was 4 years (IQR 2–7), with > 95 % completeness.

### 3.4 Primary malignant GCTB (PM-GCTB)

Five patients were diagnosed with PM-GCTB, each confirmed as a high-grade sarcoma at initial presentation, without prior benign GCTB history. Four underwent wide or en-bloc resection, one combined with chemotherapy; one additional patient received chemotherapy for metastatic disease. After a median follow-up of 11 years (IQR 2–16), one patient died of disease, one of unrelated causes, and three were alive and disease-free (Table 2).

**Table 2:** Follow up status Malignant and Metastatic Giant Cell Tumors of Bone.

Giant cell tumors of bone	Benign Pulmonary Metastatic	Primary Malignant (PM-GCTB)	Secondary Malignant (SM-GCTB)	Secondary Osteosarcoma
Number of patients	**3**	**5**	**5**	**2**
AWD %	100	0	0	0
NED %	0	60	20	100
DOD %	0	20	80	0
DOOD %	0	20	0	0

AWD = Alive With Disease, NED = No Evidence of Disease, DOD = Died Of Disease, DOOD = Died Of Other Disease.

All PM-GCTB diagnoses were made on primary resection specimens or extensive biopsies showing de novo high-grade sarcoma morphology; none had prior curettage or local recurrence. Fig. 2, Table 1, Table 2.

### 3.5 Benign pulmonary metastatic GCTB

Three patients (0.6 %) developed histologically benign pulmonary metastases after a median 3.2 years (IQR 1.3–4.4) from initial diagnosis. In all cases, the pulmonary lesions were confirmed by biopsy or VATS procedures and demonstrated typical benign GCTB morphology without sarcomatous features.

Treatment was individualized: one patient underwent VATS without further therapy, one patient received denosumab, and one patient was treated with combined chemotherapy and radiotherapy (50 Gy). All three patients remained alive with stable or slowly regressing disease at a median (median 6.7 years, IQR 2.2–9.4). Given their benign histology and indolent course, these pulmonary lesions were analyzed separately from malignant transformations (Fig. 2, Table 1, Table 2).

### 3.6 Secondary malignant GCTB (SM-GCTB)

Five patients (1.0 %) developed SM-GCTB after an initial benign diagnosis. Median latency was 7.5 years (IQR 0.6–7.5), post-primary diagnosis, with a maximum of 18 years.

In three patients, malignant recurrence occurred within the first postoperative year despite extensive curettage specimens reviewed by a tertiary bone sarcoma pathologist showing no malignant features at baseline. These cases therefore represent true, rapidly progressive biological transformation rather than a diagnostic sampling error. The remaining two cases transformed after longer latency (7 and 18 years), both following multiple recurrences. All SM-GCTB were diagnosed on resection specimens showing abrupt transition from typical GCTB areas to high-grade sarcomatous regions with atypical mitoses.

Four (80 %) developed pulmonary metastases and died of disease despite multimodal therapy (chemotherapy ± surgery) after median 0.7 years (IQR 0.6–1.8) follow-up. (Fig. 2, Table 1, Table 2). One patient underwent re-resection and adjuvant chemotherapy and remains disease-free at 1.8 years of follow-up.

This pattern highlights that, although rare, some benign GCTB can undergo unexpectedly rapid malignant progression with an aggressive biological phenotype.

### 3.7 Secondary osteosarcoma

Two patients developed secondary osteosarcoma, both arising at the site of a previously benign GCTB (latency 18 and 35 years). Both were treated with radical surgery (one amputation, one wide resection) and multiagent chemotherapy. At last follow-up (1.6 and 2.8 years, respectively), both were alive and disease-free. These extremely late transformations highlight the importance of clear patient education regarding the possibility of late recurrence or transformation, rather than routine long-term imaging surveillance.

### 3.8 Treatment exposure and confounding factors

None of the malignant or metastatic transformations occurred in patients previously treated with denosumab or radiotherapy. Across the entire cohort, 76 patients (15 %) received denosumab and 21 (4 %) underwent radiotherapy for benign disease, confirming the absence of therapy-related sarcoma genesis. Chemotherapy was administered in 40–80 % of malignant cases depending on subtype (Table 1). Recurrence and metastasis rates are detailed in Table 1 and Table 2.

### 3.9 Survival and follow-up

Disease-specific mortality differed significantly among subtypes.

Four of five SM-GCTB patients died of disease (80 %), compared with one of five PM-GCTB patients (20 %) and none of the two secondary osteosarcoma patients.

All three benign metastatic GCTB patients were alive with disease at last follow-up.

Median overall follow-up among malignant and metastatic patients was 7.2 years (IQR 2.0–15.8). Fig. 2 illustrates the individual clinical courses and survival outcomes using swimmer plots.

## 4 Discussion

This study confirms the existence of three distinct aggressive variants within the GCTB spectrum: benign pulmonary metastatic GCTB, primary malignant GCTB (PM-GCTB), and secondary malignant transformations, including secondary malignant GCTB (SM-GCTB) and secondary osteosarcoma. While PM-GCTB represents a de novo high-grade sarcoma, both SM-GCTB and secondary osteosarcoma originate from a previously benign, histologically proven GCTB. Importantly, these variants differ markedly in clinical behavior, latency, and prognosis. Patients with SM-GCTB had the poorest outcomes, with 80 % dying of disease, compared to 20 % in PM-GCTB and none in secondary osteosarcoma.

Benign pulmonary metastatic GCTB remains rare, occurring in 0.6 % of our cohort, lower than the 1–9.7 % reported in previous literature [13], [17], [18], [19], [20]. These lesions were histologically identical to benign GCTB and behaved indolently over long follow-up. Molecular studies suggest these benign pulmonary metastases result from polyclonal seeding without additional driver mutations, supporting the hypothesis that they represent embolic spread rather than true metastasis [9]. Accordingly, these cases were analyzed separately and should not be interpreted as malignant disease. Careful histopathological and radiologic evaluation is essential to avoid overtreatment.

The overall malignancy rate of 2.4 % in this study aligns with prior reports [10], [11], [21], [22]. The refined sub-classification into PM-GCTB, SM-GCTB, and secondary osteosarcoma offers valuable clinical insight, as each carries distinct implications for treatment and prognosis. PM-GCTB presents as a primary high-grade sarcoma at diagnosis, whereas SM-GCTB and secondary osteosarcoma represent malignant transformations after variable latency. Of particular note, three SM-GCTB cases transformed within the first postoperative year. All three had curettage specimens originally reviewed by a specialist bone-sarcoma pathologist and showed no malignant elements at baseline. These cases therefore represent true malignant transformation of initially benign GCTB rather than missed PM-GCTB, highlighting the unpredictable and occasionally aggressive biological behavior that can occur in a minority of GCTB.

While PM-GCTB often responds to wide excision and adjuvant chemotherapy, SM-GCTB is associated with a more aggressive and less predictable course. Secondary osteosarcoma, although rare, appears more amenable to conventional osteosarcoma regimens.

Consistent with prior studies [22], [23], patients with PM-GCTB showed better survival than those with SM-GCTB. Factors such as tumor size, extension, and treatment history influence outcomes [23], [24]. None of the malignant transformations in our cohort occurred in patients who had received radiotherapy, reducing the likelihood of confounding by radiation-induced sarcomas [25].

The diagnosis of malignant GCTB remains challenging, particularly in recurrent or atypical cases [26]. Given the evolving histopathologic criteria and sampling limitations over the past decades, accurate diagnosis increasingly depends on specialized pathological review and the integration of molecular tools. Immunohistochemical detection of H3F3A mutations (G34W or G35W) [27], [28] is particularly valuable in distinguishing GCTB and its malignant counterparts from other giant-cell-rich sarcomas and should be incorporated whenever possible.

Regarding treatment, concerns about denosumab’s role in malignant transformation remain unsubstantiated in our data, consistent with literature [29], [30], [31], [32]. Although denosumab can induce histologic changes that mimic higher-grade morphology, it remains a safe and effective option for unresectable benign GCTB when used appropriately. Vigilance is nevertheless warranted to prevent misinterpretation of treatment-related atypia [33]. Across the entire cohort, 130 patients received denosumab and 8 radiotherapy, yet no malignant transformations occurred following either therapy.

Our retrospective single-center design and long inclusion period inherently introduce variability in follow-up and diagnostic standards. However, the strengths of this study include its large cohort size, long-term follow-up, and comprehensive re-evaluation of all malignant and metastatic cases using current WHO 2020 criteria. The data provide a robust overview of the natural history of malignant GCTB and the evolution of diagnostic practice over nearly four decades. Future multicenter and molecular studies are warranted to elucidate the mechanisms driving malignant transformation and to identify predictive markers of risk. Integration of molecular profiling, radiologic characteristics, and clinical data could help refine follow-up strategies and guide early intervention.

Traditionally, some authors have proposed prolonged radiological follow-up to detect late recurrence or malignant change [34]. Based on our findings, and supported by clinical experience, such an approach is neither practical nor evidence-based. Malignant transformation is exceptionally rare and is not an asymptomatic process; all transforming cases presented with new pain or swelling. Therefore, rather than routine lifelong imaging, we advocate for thorough patient education. Patients should be counselled about the risks of recurrence and the very small chance of malignant transformation, and instructed to seek urgent evaluation by a specialist bone-tumour centre if new, unexplained, or progressive pain or swelling occurs at the previous tumour site. This pragmatic, symptom-driven strategy avoids unnecessary imaging for hundreds of patients while ensuring timely detection of clinically relevant recurrences or transformations.

In conclusion, benign pulmonary metastatic GCTB, PM-GCTB, SM-GCTB, and secondary osteosarcoma represent distinct biological and clinical entities. Both SM-GCTB and secondary osteosarcoma arise from a previously benign GCTB but diverge in morphology and prognosis. SM-GCTB carries the poorest survival, whereas secondary osteosarcoma appears to follow a more favorable course.

Malignant transformation can occur after both short and long intervals, but is exceedingly rare and is not asymptomatic. For this reason, we recommend a pragmatic, patient-centred follow-up strategy focused on comprehensive patient education rather than routine lifelong clinical or radiological surveillance. Patients should be instructed to seek prompt specialist assessment if new pain or swelling arises, ensuring early evaluation while avoiding unnecessary follow-up for the majority.

## CRediT authorship contribution statement

**Floortje G.M. Verspoor:** Writing – review & editing, Writing – original draft, Visualization, Validation, Project administration, Methodology, Investigation, Formal analysis, Data curation, Conceptualization. **Gitte G.J. Krebbekx:** Writing – original draft, Visualization, Validation. **Mylene J.C. Duivenvoorden:** Writing – review & editing, Data curation. **Vaiyapuri Sumathi:** Writing – review & editing, Supervision, Data curation. **Scott Evans:** Writing – review & editing, Supervision, Methodology, Data curation, Conceptualization.

## Funding

None.

## Declaration of competing interest

The authors declare that they have no known competing financial interests or personal relationships that could have appeared to influence the work reported in this paper.
